# Missed opportunities and barriers for vaccination: a descriptive analysis of private and public health facilities in four African countries

**DOI:** 10.11604/pamj.supp.2017.27.3.12083

**Published:** 2017-06-21

**Authors:** Comfort Zuyeali Olorunsaiye, Margaret Shaw Langhamer, Aaron Stuart Wallace, Margaret Lyons Watkins

**Affiliations:** 1 Global Immunization Division, Centers for Disease Control and Prevention, Atlanta 30329, GA, USA; 2 Emory University, Atlanta, GA, USA

**Keywords:** Immunization, vaccination, Africa, Service Provision Assessment survey

## Abstract

**Introduction:**

Missed opportunities and barriers to vaccination limit progress toward achieving high immunization coverage and other global immunization goals. Little is known about vaccination practices contributing to missed opportunities and barriers among private healthcare providers in Africa.

**Methods:**

Service Provision Assessments (SPA) of representative samples of health facilities in four African countries (Kenya, Tanzania, Senegal, Malawi) in 2010-2015 were used to describe missed opportunities and barriers for vaccination in public, private for-profit, private not-for-profit and faith-based facilities. Data included vaccination practices, observations during sick child and antenatal visits, and exit interviews following sick child visits.

**Results:**

Data from 3,219 health facilities, 11,613 sick child visits and 8,698 antenatal visits were included. A smaller proportion of for-profit facilities offered child vaccination services (country range, 25-37%) than did public facilities (range, 90-96%). The proportion of facilities offering pentavalent vaccine (diphtheria, pertussis, tetanus, hepatitis B and *Haemophilus influenza* type b antigens) daily ranged 0-77% across countries and facility types. Less than 33% of for-profit facilities in any country offered measles vaccination daily. A minority of public or private providers assessed the child’s vaccination status during a sick child visit (range by country and facility type, 14-44%), or offered tetanus toxoid during antenatal visits (range, 19-51%). Very few providers discussed the importance of newborn vaccination.

**Conclusion:**

Substantial missed opportunities for, and barriers to, vaccination were identified across this representative sample of health facilities in four African countries. Strategies are needed to ensure that private and public providers implement practices to minimize barriers and missed opportunities for vaccination.

## Introduction

In many low and middle-income countries (LMICs), immunization services are provided solely through government-funded public providers, but recent evidence indicates the private health sector may also play an important role in immunization service provision [1]. The Global Vaccine Action Plan 2011-2020 (GVAP) proposes increased private sector involvement in community demand and related vaccination coverage improvement strategies to *“ensure strong immunization systems are an integral part of a well-functioning health system”* [2].

Little is known about the characteristics of private sector delivery of immunization services in LMICs, including how private and public sector practices differ along lines of service quality and adherence to national immunization policies [1]. The current limited evidence indicate private healthcare providers have lower levels of knowledge about immunization and service quality compared to public sector providers [1, 3, 4]; however only one published study from Africa is currently available [4].

The GVAP calls on all countries to reach ≥ 90% national coverage and ≥ 80% coverage in every district for all vaccines in the country’s national immunization schedule by 2020 [2]. Global coverage, as measured by the third dose of diphtheria-tetanus-pertussis vaccine (DTP3), has remained stagnant at 85% since 2010 [5]. Of particular concern is the World Health Organization (WHO) African Region, which had the lowest DTP3 coverage (76%) of all WHO regions in 2015.

Minimizing missed opportunities for vaccination (MOV) is one important strategy to reach and sustain ≥ 90% coverage. MOV occur when an individual presents for any health service and is not provided all vaccinations for which they are eligible. Every preventive and curative health service encounter is an opportunity to vaccinate an eligible individual. MOV are common; a 2014 review in LMICs concluded that MOV occurred in 32%–46% of public health service encounters [6]. Recent assessments in Africa reported high MOV prevalence, ranging from 43% to 57%, and documented multiple reasons for an MOV [7, 8], including healthcare providers not checking a child’s vaccination eligibility during a sick child visit, false contraindications for vaccination, and hesitancy to open multi-dose vaccine vials. Given this evidence, in 2016 the WHO Strategic Advisory Group of Experts on Immunization endorsed a global strategy to identify and reduce MOV prevalence worldwide [8, 9].

Strategies for reducing MOV include screening for vaccination status during visits to health facilities for mild illness, ensuring children receive all recommended vaccinations during a single visit, and using facility-based child vaccination registers to help track when individuals are due for vaccination [10]. Both private and public health sectors could play important roles in implementing MOV reduction strategies. Although MOV have been examined in the public sector, little is known about MOV in the private health sector.

Health sector surveys such as Service Provision Assessment (SPA) surveys provide an opportunity for insight into MOV across private and public sectors. SPA surveys are conducted every 3-7 years in LMICs to assess availability, delivery, and quality of services in a representative sample of health facilities. This paper assesses potential barriers and MOV in public and private (for-profit, not-for-profit and faith-based) health facilities in four African countries that conducted SPA surveys during 2010-2015: Kenya, Tanzania, Senegal and Malawi.

## Methods

SPA surveys collect information on facility-based health services in developing countries using service readiness indicators developed by WHO and the United States Agency for International Development (USAID) [11, 12]. Ten health services and topics are assessed in SPA surveys. We used data from two components of SPA surveys: 1) facility inventory assessments (at all sampled health facilities), and 2) sick child and antenatal care visit observations and exit interviews (from among all sampled health facilities where visits were occurring on the day the inventory assessment occurred). We assessed potential barriers to services, including service availability, fees, and satisfaction with care. We also assessed MOV during sick child visits when recommended childhood vaccinations should be provided to eligible children, and during antenatal care visits when tetanus toxoid (TT) should be provided to eligible pregnant women.

### SPA survey methodology

Typically, SPA surveys collect data from a nationally representative sample of 400-700 formal sector health facilities [12]. In Kenya, Tanzania, and Senegal, the sample of health facilities is designed to be representative at the national and regional levels by type of facility and by the different operating authorities. In Malawi, a census of all formal-sector health facilities was conducted.

Generally, SPA surveys include 10-15 survey teams, each comprised of 3-4 surveyors. During the facility visit, the facility manager, person in-charge of the facility, or the most senior health worker is interviewed using the facility inventory questionnaire to obtain information on availability of health services, resources for these services and procedures followed for providing these services.

Surveyors also observe client-provider consultations for a single day using standardized observation protocols for antenatal care, curative care for sick children, and other services [12, 13]. SPA surveys do not include observations or exit interviews following well child or immunization visits. Surveyors attempt to select two new antenatal clients for every follow-up client. Only children under five years of age who present with an illness (as opposed to an injury or an eye or skin infection only) are selected. Where many eligible clients are present, the surveyors select no more than five clients per provider and no more than 15 clients for a particular service in any health facility [14-16].

Service observations are coupled with exit interviews using standardized questionnaires to document the client’s experience with the services received. For sick child visits, the child’s caregiver (usually a parent) participates in the exit interview. The surveyor reads a list of common topics associated with client dissatisfaction, asking if each topic posed a major or minor problem, or no problem during the visit.

### Definitions

Health facility type was defined by operating authority categories used in SPA surveys: 1) public; 2) private for-profit (for-profit), 3) private not-for-profit/non-governmental organization (not-for-profit); and 4) private mission/faith-based organization (faith-based). In Tanzania, parastatal facilities were categorized as private not-for-profit facilities per Tanzania Ministry of Health definitions [17].

### Data inclusion criteria

SPA surveys conducted during 2010-2015 in African countries (Kenya, Tanzania, Senegal and Malawi) were included in the analyses, using facility-level (inventory) and individual-level (observation protocol and client exit interview) questionnaire data [14-16, 18]. All facilities interviewed in the SPA surveys were included in our facility-based analyses unless they offered only inpatient services or stand-alone voluntary counseling and testing or used different inventory questionnaires for a specific facility type, such as “health huts” in Senegal (community-based outreach site run by community health workers). Multiple client exit interviews and observation protocols were conducted at the surveyed facilities. All sick child exit interviews and observations and antenatal observations from the sampled facilities were included unless clients refused.

In the Kenya SPA (2010) survey, 703 facilities were randomly sampled from a master list of 6,192 facilities; due to non-response, 695 facilities were included in the final dataset [14]. For the Tanzania SPA (2014-15) survey, of 7,102 verified health facilities, 1,200 were sampled and 1,188 were included in the final dataset [16]. Senegal conducts a continuous SPA survey each year covering various health service areas. Facility-level data from the 2012-13 survey were analyzed; of 3,084 health facilities, 458 were sampled and 438 successfully interviewed [18]. Observations and exit interviews for sick child visits in Senegal were available from facilities surveyed in 2012-13; antenatal care observation data were available for 2014 from a different set of facilities. Analyses accounted for weights to account for the probability of selection of facilities within defined strata (region, facility type (e.g. clinic, hospital) and operating authority), and adjusted for non-response [12]. The Malawi SPA (2013-14) survey was designed as a census of all 1,060 formal-sector health facilities in the country; 977 were included in the final dataset. The Malawi data were weighted for non-response [15].

### Data analysis

For Kenya, Tanzania, and Senegal, sample strata were created by crossing region and facility type. When a single unit stratum was present, adjacent strata from the same region were collapsed to calculate sampling errors. This was done separately for facility- and individual-level analyses since some facilities did not have any individual-level observations, resulting in a greater number of single unit strata than at the facility level. At the individual-level, facility was treated as the primary sampling unit. Since Malawi conducted a census of facilities, facility-level observations were not stratified. Individual-level observations were stratified by facility.

Each individual-level sick child observation and exit interview was merged with the inventory questionnaire data for the facility at which that observation was conducted. For questions regarding TT receipt or counseling about newborn vaccination, each antenatal care observation was merged with the inventory questionnaire data for the facility at which that observation was conducted.

For availability of vaccination services, supply of immunization cards, and service fees, data from the facility inventory questionnaire were used. Pentavalent vaccine (combination vaccine with diphtheria, pertussis, tetanus, hepatitis B and *Haemophilus influenza* type b antigens) was used as a proxy for general vaccination service delivery. Daily vaccination was defined as provision of vaccination at least 20 days per month. For assessment of caregiver perception of service quality and satisfaction, data from the sick child exit interviews were used. For measured wait times, data from sick child visit observations were used. Percentages were not calculated for facility types within a country if there were ≤ 5 facilities or interviews within that facility type.

To assess MOV among children, we used data from observed sick child visits to assess if the provider reviewed child’s health card, and assessed immunization status (i.e., if provider looked at the child’s health card either before beginning the consultation, or while collecting information from the caretaker, or while examining the child). We used data from the provider’s response when asked *“Did you* ***vaccinate*** *the child during this visit or refer the child for* ***vaccination*** *today other than vitamin A supplementation? If no: Why not?”*, to assess if the provider reported vaccinating or referring the child for vaccination or the reason for not doing so. To assess MOV among pregnant women we used data from observed antenatal visits to assess if the provider administered or prescribed TT vaccination and provided counseling about the importance of newborn vaccination.

We used the survey command in STATA 12, with the provided survey weights from the SPA survey dataset for our analyses, accounting for stratification and clustering as described above. We calculated weighted proportions for each indicator of interest across all facilities in each country and by facility type. We conducted chi-square tests to assess whether the frequency of an indicator significantly differed across the four facility types, and t-tests for pairwise analyses comparing public facilities with each of the other three facility types when the chi-square test indicated overall significance. We used a p-value threshold of ≤ 0.05 to assess statistical significance. For Malawi facility-level indicators only, no statistical tests of significance were conducted since the total number of facilities per strata were not available to calculate a finite population correction to ensure a proper estimate of variance for each indicator.

## Results

The study included data from 3,219 surveyed facilities in the four countries (Table 1). In Kenya, 5 facilities were excluded because they offered only inpatient services or stand-alone voluntary counseling and testing. In Senegal, 74 “health hut” facility types were excluded because different inventory questionnaires were used for those facilities. With the exception of Tanzania, the majority of the private facilities were for-profit facilities.

**Table 1:** Characteristics of health facilities with Service Provision Assessment (SPA) surveys, by facility type, in Kenya 2010), Tanzania (2014-2015), Senegal (2012-2013, 2014), and Malawi (2013-2014)

Country	Facility indicator	Public N (%)	Private for-profit N (%)	Private not-for-profit/NGO N (%)	Mission/faith based N (%)	Total N (%)
*Kenya*	Total facilities surveyed	345 (50)	215 (34)	38 (3)	92 (13)	690
	Facilities providing sick child care	334 (98)	196 (94)	21 (87)	87 (98)	638 (96)
	Facilities providing antenatal care	320 (88)	140 (46)	17 (77)	81 (88)	560 (81)
	Sick child visit observations	1452	260	57	247	2016 (520^5^)
	Antenatal visit observations	1030	151	25	206	1412 (396^5^)
*Tanzania^2^*	Total facilities surveyed	780 (72)	184 (14)	20 (2)	204 (12)	1188
	Facilities providing sick child care	776 (100)	161 (91)	18 (71)	199 (97)	1154 (98)
	Facilities providing antenatal care	754 (96)	80 (36)	12 (17)	185 (81)	1031 (85)
	Sick child visit observations	3723	364	50	824	4961 (1015^5^)
	Antenatal visit observations	2906	249	34	818	4007 (815^5^)
*Senegal*	Total facilities surveyed^3^	305 (83)	43 (12)	1 (0)	15 (5)	364
	Facilities providing sick child care^3^	298 (98)	28 (64)	1 (100)	15 (100)	342 (94)
	Facilities providing antenatal care^4^	290 (99)	29 (73)	11 (87)	19 (100)	349 (96)
	Sick child visit observations^3^	1118	102	4	83	1307 (328^5^)
	Antenatal visit observations^4^	1096	44	35	36	1211 (300^5^)
*Malawi*	Total facilities surveyed	478 (48)	275 (29)	57 (6)	167 (17)	977
	Facilities providing sick child care	457 (95)	249 (90)	49 (86)	165 (99)	920 (94)
	Facilities providing antenatal care	408 (85)	72 (26)	10 (17)	153 (91)	643 (65)
	Sick child visit observations	2173	461	94	601	3329 (748^5^)
	Antenatal visit observations	1487	72	18	491	2068 (412^5^)
Overall	Total facilities surveyed	1908	717	116	478	3219
	Total sick child visit observations	8466	1187	205	1755	11613
	Total antenatal care observations	6519	516	112	1551	8698

1 Senegal SPA survey collects subset of data annually instead of all data in one year; sick child data available for 2012-2013; antenatal care data available for 2014.

2 Tanzania categorized operating authority as public, private for-profit, parastatal, or mission/faith-based. Parastatal facility data shown in private not-for-profit column.

3 Refers to facilities/observations included in the 2012-2013 SPA survey.

4 Refers to facilities/observations included in the 2014 SPA survey.

5 Number of facilities within country where these observations occurred.

Most facilities in the four countries provided sick child and antenatal care; a lower proportion of for-profit and not-for-profit facilities than public facilities provided antenatal care (Table 1). We included data from 11,613 sick child visit observations and interviews, and 8,698 antenatal visit observations. There were 31 refusals for sick child interviews in Kenya, 1 in Tanzania, none in Senegal and 112 in Malawi. For antenatal interviews, there were 33 refusals in Kenya, 3 in Tanzania, none in Senegal and 37 in Malawi. Most but not all of the facilities that provided sick child or antenatal care contributed sick child or antenatal observations (Table 1).

### Potential barriers to immunization services

#### Availability of immunization services and immunization cards

In all countries, higher proportions of public (country range, 91%-96%) facilities offered childhood immunization services than did for-profit (country range, 25%-37%) facilities (Table 2). In Tanzania, a higher proportion of public facilities offered childhood immunization services than did all three other facility types. The proportion of facilities offering pentavalent vaccine daily ( ≥ 20 days/month) varied from none to 77% across countries and facility types (Table 2). In Kenya, a higher proportion of public facilities offered pentavalent vaccine daily, compared to other facility types.

**Table 2:** Characteristics of immunization services at facilities, by facility type - Service Provision Assessment (SPA) surveys in Kenya (2010), Tanzania (2014-2015), Senegal (2012-2013), and Malawi (2013-2014)

	Public %	Private for-profit % (p value)^1^	Private not-for-profit/NGO % (p value)^1^	Mission/Faith-based % (p value)^1^	p value^2^
**Facility characteristic**			***Kenya (N=690)***		
Offers immunization service	91	37 (<0.01)	75 (0.08)	80 (0.18)	<0.01
Offers daily (≥20 days/month) pentavalent vaccination	64	37 (<0.01)	37 (0.03)	47 (0.02)	<0.01
Offers daily measles vaccination	51	23 (<0.01)	35 (0.16)	29 (<0.01)	<0.01
Health cards available^3^	92	87	87	89	0.74
Client fee for immunization service	20	75 (<0.01)	43 (0.18)	81 (<0.01)	<0.01
			***Tanzania*^4^*(N=1188)***		
Offers immunization service	95	27 (<0.01)	16 (<0.01)	79 (<0.01)	<0.01
Offers daily (≥20 days/month) pentavalent vaccination	69	63	69	77	0.41
Offers daily measles vaccination	24	11	20	28	0.42
Health cards available^3^	87	91	87	76	0.07
Client fee for immunization service	1	1	0	3	0.48
			***Senegal*^5^*(N=364)***		
Offers immunization service	93	30 (<0.01)	-	79 (0.22)	<0.01
Offers daily (≥20 days/month) pentavalent vaccination	40	44 (0.78)	-	0 (<0.01)	0.02
Offers daily measles vaccination	5	33 (0.22)	-	0 (<0.01)	<0.01
Health cards available^3^	89	86	-	87	0.93
Client fee for immunization service	36	34	-	12	0.27
			***Malawi*^6^*(N=977)***		
Offers immunization service	96	25	33	95	-
Offers daily (≥20 days/month) pentavalent vaccination	43	13	26	46	-
Offers daily measles vaccination	40	8	21	41	-
Health cards available^3^	57	46	42	58	-
Client fee for immunization service	0	7	5	3	-

1 p value (t-test) for pairwise difference in proportion compared to public facilities; test done only among those indicators where the overall chi-square test result indicated a significant difference between any facility types.

2 Chi square p-values for differences across all facility types.

3 Of facilities offering immunization services.

4 Tanzania categorized operating authority as public, private-for-profit, parastatal, or mission/faith-based. Parastatal facility data shown in private not-for-profit column.

5 Percentages not calculated for private not-for-profit category because only 1 private not-for-profit facility surveyed in Senegal.

6 No facility level statistical tests done for Malawi due to census design.

**Figure 1:**
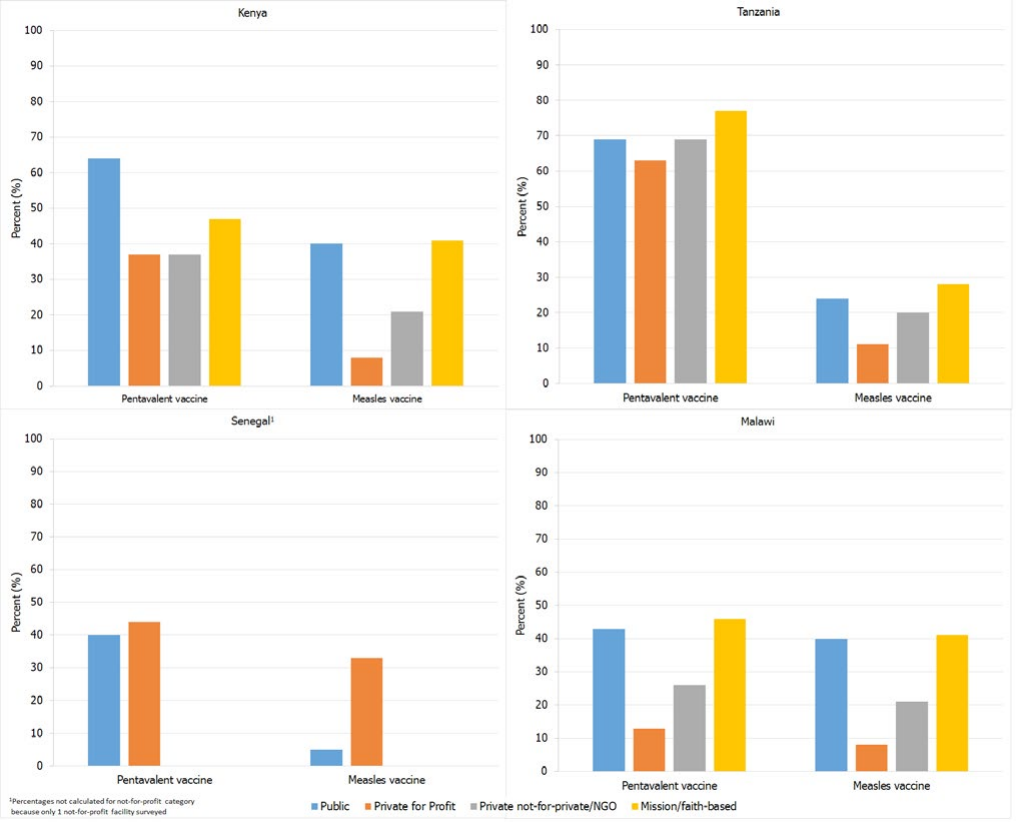
Proportion of facilities offering pentavalent and measles vaccines on a daily (≥20 days/month) basis, by facility type – Service Provision Assessment surveys in Kenya (2010), Tanzania (2014-2015), Senegal (2012-2013), and Malawi (2013-2014)

Among for-profit facilities, 8%-33% (country range) offered measles vaccine daily compared to 13%-63% that offered pentavalent vaccine daily (Table 2, Figure 1). A majority of each facility type in each country had a supply of blank immunization cards except for those in Malawi where only about half of all facilities (facility type range, 42%-58%) had blank immunization cards available (Table 2).

#### Fees for immunization services

The proportion of facilities that reported charging fees for immunization visits varied among countries. In Tanzania and Malawi, few facilities of any operating authority type (facility type range, 0%-3% for Tanzania, 0%-7% for Malawi) charged fees for immunization visits (Table 2). In Kenya, a higher proportion of for-profit (75%) and faith-based private facilities (81%) reported that they charged fees for immunization visits than did public facilities. However, a substantial proportion of public facilities in both Kenya (20%) and Senegal (36%) charged fees for immunization visits. In Kenya, additional questions were asked about why fees were charged: of the public facilities in Kenya that charged fees for immunization services, 66% charged for the immunization card, 26% for vaccine administration and 13% for vaccine.

### Missed opportunities for vaccination

#### During sick child visits

The proportion of providers who were observed reviewing the child health card for any reason during a sick child visit varied widely by country and facility type (Table 3). A lower proportion of providers in for-profit facilities in Tanzania (21%) and Malawi (67%) than in public facilities in Tanzania (42%) and Malawi (76%) reviewed the child health card. In Malawi a higher proportion of providers in not-for-profit (82%) and faith-based facilities (84%) than in public facilities (76%) reviewed the card. Specific assessment of the child’s immunization status was less commonly observed; a minority of providers in all countries and all facility types did so (Table 3). In Malawi, a lower proportion of public providers assessed vaccination status compared to for-profit, not-for-profit and faith-based providers.

**Table 3:** Service characteristics observed during sick child visit or antenatal visit, by facility type - Service Provision Assessment (SPA) surveys in Kenya (2010),Tanzania (2014-2015), Senegal^1^ (2012-2013, 2014), and Malawi (2013-2014)

	Public % (p value)^2^	Private for- profit % (p value)^2^	Private not-for-profit/NGO % (p value)^2^	Mission/Faith-based % (p value)^2^	p value^3^
***Kenya***				
***Sick child visit (N=2016)***					
Provider reviewed child health card^6^	80	66	81	69	0.29
Provider assessed immunization status^6^	44	32	34	34	0.37
Vaccination administered^7^	7	3 (0.03)	39 (0.06)	7 (0.88)	<0.01
Child referred for vaccination^7^	4	2	2	2	0.51
Child “not due”/Fully immunized^7^	68	69	44	58	0.24
Not immunization day^7^	2	3	2	4	0.60
No immunization check^7^	17	19	11	26	0.40
Antenatal visit (N=1412)					
TT administered/prescribed^6^	51	42	45	48	0.55
Newborn vaccination discussed^6^	13	3	15	23	0.11
	***Tanzania ^4^***				
***Sick child visit (N=4961)***					
Provider reviewed child health card^6^	42	21 (<0.01)	21 (0.19)	38 (0.39)	<0.01
Provider assessed immunization status^6^	25	18	15	23	0.36
Vaccination administered^7^	3	0	2	2	0.20
Child referred for vaccination^7^	0	1	0	0	0.90
Child “not due”/Fully immunized^7^	61	65	58	59	0.69
Not immunization day^7^	21	11	23	22	0.07
No immunization check^7^	13	9	16	16	0.35
Antenatal visit (N=4007)					
TT administered/prescribed^6^	36	38	19	35	0.75
Newborn vaccination discussed^6^	3	2	0	3	0.95
	***Senegal***				
***Sick child visit^5^ (N=1307)***					
Provider reviewed child health card^6^	14	33 (0.08)	-	41 (<0.01)	<0.01
Provider assessed immunization status^6^	27	18	-	17	0.16
Vaccination administered^7^	4	6	-	1	0.47
Child referred for vaccination^7^	1	0	-	0	0.63
Child “not due”/Fully immunized^7^	27	14 (<0.01)	-	7 (<0.01)	<0.01
Not immunization day^7^	18	19	-	22	0.70
No immunization check^7^	49	60	-	70	0.06
Antenatal visit (N=1211)					
TT administered/prescribed^6^	33	47	24	37	0.27
Newborn vaccination discussed^6^	0	0	0	3	0.48
	***Malawi***				
***Sick child visit (N=3329)***					
Provider reviewed child health card^6^	76	67 (<0.01)	82 (<0.01)	84 (<0.01)	<0.01
Provider assessed immunization status^6^	14	22 (<0.01)	30 (<0.01)	23 (<0.01)	<0.01
Vaccination administered^7^	2	1 (<0.01)	7 (0.10)	2 (0.89)	<0.01
Child referred for vaccination^7^	2	1	2	1	0.66
Child “not due”/Fully immunized^7^	61	57 (0.02)	49 (0.02)	62 (0.64)	0.02
Not immunization day^7^	6	5 (0.05)	16 (<0.01)	10 (<0.01)	<0.01
No immunization check^7^	28	35 (<0.01)	17 (<0.01)	25 (0.04)	<0.01
Antenatal visit (N=2068)					
TT administered/prescribed^6^	26	24 (0.66)	50 (0.03)	38 (<0.01)	<0.01
Newborn vaccination discussed^6^	2	2 (0.96)	26 (<0.01)	3 (0.04)	<0.01

1 Senegal SPA collects subset of data annually; sick child data available for 2012-2013; antenatal care data available for 2014.

2 p value (t-test) for pairwise difference in proportion compared to public facilities; test done only among those indicators where the overall chi-square test result indicated a significant difference between any facility types.

3 Chi square p-value for differences across facility types.

4 Tanzania categorized operating authority as public, private for profit, parastatal, or mission/faith-based. Parastatal facility data shown in private not-for-profit column.

5 Percentages not calculated because <5 (n=4) sick child interviews in private not-for-profit facilities in Senegal.

6 Observed by surveyor

7 Reported by provider

During sick child visits, few providers reported administering vaccinations or referring the child for vaccination (Table 3). Rather, providers indicated that the child was not due for vaccination, that they had not checked the child’s vaccination status, or that the sick child visit occurred on a non-immunization day.

#### During antenatal care visits

In each country, ≤ 51% of providers in any facility type administered or prescribed TT to pregnant women (Table 3). Overall, very few providers, in public or private facilities, discussed the importance of newborn vaccination with the pregnant woman (country range, 0-15%).

### Health facility services and caregiver satisfaction

#### Wait times

Compared to other facility types, observed mean and median wait times for sick child visits were the shortest in for-profit facilities in each country except Senegal. For example, median wait times were 15, 30 and 10 minutes in for-profit facilities, compared to 30, 60, and 80 minutes in public facilities in Kenya, Tanzania and Malawi respectively (data not shown). In Kenya and Malawi, approximately 25% of caregivers visiting public facilities reported that wait times were a major problem, the highest proportion among the facility types (Table 4).

**Table 4:** Caregivers’ satisfaction with sick child services, by facility type - Service Provision Assessment (SPA) surveys in Kenya (2010), Tanzania (2014-2015), Senegal (2012-2013), and Malawi (2013-2014)

Caregiver responses	Public % (p value)^2^	Private for profit % (p value)^2^	Private not-for-profit/NGO^1^ % (p value)^2^	Mission/Faith-based % (p value)^2^	p value^3^
	*Kenya (N=2016)*				
***Reported major problems***					
Wait time	28	12 (<0.01)	10 (<0.01)	13 (<0.01)	<0.01
Availability of medicine	21	2 (<0.01)	32 (0.48)	1 (<0.01)	<0.01
Hours of service	9	8	10	2	0.29
Days of service	6	2	11	2	0.10
Facility cleanliness	2	1	1	1	0.14
Provider attitude	2	0	1	0	0.35
Very satisfied with services	74	76	82	76	0.79
Not satisfied with services	3	0 (<0.01)	3 (0.26)	0 (<0.01)	<0.01
Facility visited not closest to home	14	34 (<0.01)	7 (0.09)	37 (<0.01)	<0.01
	***Tanzania (N=4961)***				
***Reported major problems***					
Wait time	16	11	19	17	0.53
Availability of medicine	20	5 (<0.01)	16 (0.66)	7 (<0.01)	<0.01
Hours of service	6	3	2	2	0.09
Days of service	3	1	1	1	0.06
Facility cleanliness	5	2 (0.02)	1 (<0.01)	1 (<0.01)	<0.01
Provider attitude	2	1	1	2	0.77
Very satisfied with services	74	74	82	75	0.85
Not satisfied with services	6	3	0	4	0.41
Facility visited not closest to home	11	39 (<0.01)	29 (0.09)	31 (<0.01)	<0.01
	***Senegal ^4^(N=1307)***				
***Reported major problems***					
Wait time	15	33	-	20	0.16
Availability of medicine	10	10	-	3	0.23
Hours of service	6	11	-	9	0.62
Days of service	3	0 (<0.01)	-	11 (0.08)	<0.01
Facility cleanliness	3	0	-	0	0.61
Provider attitude	1	6	-	2	0.18
Very satisfied with services	82	89	-	74	0.08
Not satisfied with services	0	0	-	0	0.90
Facility visited not closest to home	18	65 (<0.01)	-	54 (<0.01)	<0.01
	***Malawi (N=3329)***				
***Reported major problems***					
Wait time	25	8 (<0.01)	10 (<0.01)	15 (<0.01)	<0.01
Availability of medicine	16	5 (<0.01)	2 (<0.01)	4 (<0.01)	<0.01
Hours of service	14	2 (<0.01)	2 (<0.01)	6 (<0.01)	<0.01
Days of service	8	2 (<0.01)	7 (0.47)	3 (<0.01)	<0.01
Facility cleanliness	5	1 (<0.01)	2 (0.05)	1 (<0.01)	<0.01
Provider attitude	5	1 (<0.01)	2 (0.07)	2 (<0.01)	<0.01
Very satisfied with services	81	81	83	82	0.90
Not satisfied with services	6	3 (<0.01)	1 (<0.01)	3 (<0.01)	<0.01
Facility visited not closest to home	10	36 (<0.01)	28 (<0.01)	24 (<0.01)	<0.01

1 Tanzania categorized managing authority as public, private for profit, parastatal, or mission/faith-based. Parastatal facility data shown in private not-for-profit column.

2 p value (t-test) for pairwise difference in proportion compared to public facilities; test done only among those indicators where the overall chi-square test result indicated a significant difference between any facility types.

3 Chi square p-value for differences across operating authority types

4 Percentages not calculated because <5 (n=4) sick child interviews in private not-for-profit facilities in Senegal.

#### Other services

In Kenya, Tanzania and Malawi, a higher proportion of caregivers at public facilities reported major problems with availability of medicine compared to caregivers at for-profit or faith-based facilities (Table 4). Only a small proportion of caregivers (<5%) in any country reported major problems with facility cleanliness or provider attitude, regardless of facility type (Table 4). However, in Tanzania and Malawi, caregivers at public facilities more often reported problems with facility cleanliness compared to caregivers at other facility types; caregivers at Malawi public facilities reported problems with provider attitude more than in other facility types. The proportion of caregivers reporting a major problem with hours or days of service delivery was low (<14%) in all facility types in all four countries; in Malawi problems with hours or days of service reported by caregivers were higher in public facilities than in for-profit or faith-based facilities.

#### Caregiver satisfaction

The majority (facility type range, 74%-83%) of clients in each country reported being very satisfied with services, regardless of facility type. The proportion of caregivers not satisfied with services was low (<6% in all countries and facility types), but in Kenya and Malawi it was higher in public facilities than in for-profit or faith-based facilities. A notable proportion of caregivers sought sick child services at a facility other than the one closest to their home. Of those, more sought care at for-profit facilities (country range, 34-65%) or faith-based facilities (country range, 24%-54%), compared to public facilities. The most common reasons for not visiting the facility closest to their residence included the closer facility being too expensive (country range 5%-20%), having a bad reputation (country range, 4-19%), or having no medication (country range, 3%-21%), or the caregiver received a referral to the farther clinic (country range, 5%-21%).

## Discussion

This study was the first to use SPA surveys to compare factors related to vaccination barriers and MOV across public and private health facilities. Data were drawn from nationally representative surveys, whose large sample sizes improve generalizability of results. The high facility response rates (99%) and the use of standardized questionnaires across countries enabled transnational comparisons. We observed differences in barriers to vaccination by facility type, including less availability of child immunization services in for-profit compared to public facilities in all countries, a higher proportion of for-profit facilities charging fees than public facilities in Kenya, and higher proportions of caregivers reporting problems with wait times and unsatisfactory care in public facilities than in for-profit facilities in Kenya and Malawi. We identified common missed opportunities across facility types, including infrequent vaccination screening and vaccine administration during sick child visits, and infrequent TT administration and counseling about newborn vaccination during antenatal care visits. This study helps to fill a knowledge gap about vaccination practices in the private sector and identifies potential opportunities to increase access to and utilization of immunization services across all facility types.

Fees for immunizations, whether official or informal, create an important barrier to vaccinations [19], and their existence is particularly concerning in public facilities. Although user fees were uncommon in Tanzania and Malawi, a substantial proportion of public facilities charged fees in Kenya and Senegal. Understanding how and why public facilities charge user fees for immunization services is an important future step for addressing how to remove this barrier to vaccination. Of the four countries in this study, only the Kenya data included information on specific fees charged for immunization services. More information from additional countries is needed.

Wait times were reported as a major problem across all types of facilities in all four countries. Long wait times are often cited as a barrier to health care seeking [19, 20]. Private facilities were found to have shorter wait times, but also were more likely to charge fees and many offered immunization services at limited times or not at all.

Despite problems that caregivers reported with services they received, the majority, across all facility types, reported satisfaction with services, similar to findings from other studies that reported relatively high levels of client satisfaction with the quality of primary health care services received [21, 22]. Clients are more likely to adhere to recommended care if they are satisfied with the quality of services [23]. Thus concerted efforts are needed to ensure that immunization services are of high quality and acceptable to clients. Monitoring for key barriers such as user fees and long wait times would enable these issues to be addressed in a timely manner to maintain client satisfaction.

A significant proportion of providers across countries and facility types did not screen sick children for eligible vaccinations, and vaccination was uncommon during sick child visits. Immunization histories of the sick children and their illness severity are unknown, so it is not possible to ascertain the proportion of children eligible for vaccination who were not screened. A recent study in Dominican Republic [24] found that 53% of children visiting health facilities for illness and eligible for vaccination had MOV, compared to 40% of children visiting for vaccination. A review of 57 studies of MOV [6] found a pooled MOV prevalence among children of 32.2%; the most common reasons for MOV related to health care provider practices, of which not reviewing immunization cards or vaccination history were the most common.

Additionally, we identified a potential MOV specific to measles vaccination; the majority of surveyed facilities, particularly for-profit facilities, reported providing measles vaccination less frequently than pentavalent vaccination. This practice may stem from the requirement that a measles vaccine vial be discarded within six hours of being opened, unlike pentavalent vaccine which can be stored for later use. Providers may be reluctant to open a multi-dose measles vaccine vial when only a few children present for vaccination; instead the child is told to return on a special measles vaccination day [25, 26]. Turning away a child creates an MOV, with uncertainty that the child will return, particularly if the caregiver has to travel far to the facility. Availability of measles vaccines containing fewer doses, for example five-dose vials, may contribute to reducing MOVs caused by reluctance to open a ten-dose measles vaccine vial for a few children.

Pregnant women receiving antenatal care often did not receive TT. Thus, most providers missed an important opportunity to protect neonates from tetanus at birth. TT vaccination contributes significantly to the elimination of neonatal tetanus. Across all facility types, there is a need to educate or re-orient providers on the importance of TT for pregnant women. Antenatal visits also provide an opportunity to increase awareness about newborn immunization, yet our results indicate such education rarely occurred. It is important for policy makers and service providers to make use of the opportunity that antenatal visits offer to improve maternal and child health. Further, an additional MOV for TT vaccination for women exists when women bringing their infants for routine immunization or sick child visits are not screened and vaccinated as indicated.

### Limitations

Some facilities did not provide sick child or antenatal care on the day of the surveys, so observations and exit interviews were not conducted there. The characteristics of these facilities are unknown. However, of facilities that provided sick child or antenatal care, the majority contributed sick child observations (85%) or antenatal care observations (74%), likely minimizing potential bias. Variations in country context relative to health systems (including private provider practices) and health care seeking behavior may complicate country comparisons and limit generalizability to other LMICs.

We used exit interviews following sick child visits to assess caregiver perceptions of care. Assessments following immunization visits may have yielded more specific information about provider immunization practices, but were not available in SPA surveys. The method of conducting exit interviews at the health facility may predispose clients to give socially desirable answers, resulting in higher reported satisfaction, but the addition of structured observation may mitigate some of this potential bias. Our analysis focused on generating hypotheses for future studies and was not intended to assess causal associations. To do so would require study designs incorporating collection and analysis of applicable covariate data alongside exposure and outcome data. Not all countries participate in SPA surveys and they are infrequent, limiting broader analysis of SPA survey data.

## Conclusion

Despite substantial progress in increasing the number of children vaccinated worldwide, over a third of all countries have not yet met the Global Vaccine Action Plan 2011–2020 target of 90% national DTP3 coverage [5]. We found potential barriers and missed opportunities for vaccination in public and private (for-profit, not-for-profit, and faith-based) health facilities in four sub-Saharan African countries. Several barriers and missed opportunities for vaccination were more prevalent in private facilities than in other facility types. Potential interventions include those that address needs for training and guidance; structural and systems changes such as policies for vaccination screening and removal of fees; and specific interventions to engage the private sector in delivery of high quality vaccination services. Reducing measles vaccine vial size may contribute significantly to reducing measles MOVs given the concerns of vaccine wastage which, in most cases, lead to health facilities having special days for measles vaccinations. In some countries, working through professional societies may provide opportunities to incorporate immunization training into professional development standards and to communicate vaccination policies. Minimizing missed opportunities and barriers to vaccination in both public and private health sectors can help to improve immunization coverage to meet development goals.

### What is known about this topic

- The African region has the lowest immunization coverage of all WHO regions (76% DPT3 coverage in 2015);
- Reducing missed opportunities for and barriers to vaccination can improve immunization coverage and reduce immunity gaps;
- There is limited information about immunization practices in private health facilities; very few studies have compared immunization practices in public and private facilities in low and middle income countries.

### What this study adds

- Service provider assessments provide useful large-scale and representative information about immunization in both public and private health facilities, and enabled identification of substantial missed opportunities and barriers to vaccination in four African countries;
- A smaller proportion of for-profit health facilities than public facilities in four African countries offer childhood immunization services. Less than a third of for-profit facilities offered measles vaccination daily;
- A minority of public and private providers in four African countries assessed immunization status of children during sick child visits, offered tetanus toxoid vaccine to pregnant women, or discussed the importance of newborn vaccination during antenatal visits.

## Competing interests

The authors declare no competing interest.
